# Succession and Driving Factors of Periphytic Community in the Middle Route Project of South-to-North Water Division (Henan, China)

**DOI:** 10.3390/ijerph19074089

**Published:** 2022-03-30

**Authors:** Xiaonuo Chen, Xiaojun Wang, Yuying Li, Yinlei Yao, Yun Zhang, Yeqing Jiang, Xiaohui Lei, Han Liu, Naicheng Wu, Nicola Fohrer

**Affiliations:** 1 International Joint Laboratory of Watershed Ecological Security and Collaborative Innovation Center of Water Security for Water Source Region of Middle Route Project of South-North Water Diversion in Henan Province, College of Water Resource and Environment Engineering, Nanyang Normal University, Nanyang 473061, China; chenxn1996@126.com (X.C.); yyl19892021@163.com (Y.Y.); zy174812@163.com (Y.Z.); jyq61359@163.com (Y.J.); liuhan2008@163.com (H.L.); naichengwu88@gmail.com (N.W.); nfohrer@hydrology.uni-kiel.de (N.F.); 2 Qushou Branch Bureau of Construction and Administration Bureau of South-to-North Water Division Project, Nanyang 473000, China; wmdgs09@163.com; 3 State Key Laboratory of Simulation and Regulation of Water Cycle in River Basin, China Institute of Water Resources and Hydropower Research, Beijing 100038, China; lxh@iwhr.com; 4 Department of Hydrology and Water Resources Management, Kiel University, 24098 Kiel, Germany

**Keywords:** periphytic algae, community structure, environmental factors, GAM

## Abstract

The Middle Route Project of the South-to-North Water Diversion is an artificially independent system that does not connect to other surface waters. Excessive periphyton proliferation causes a series of environmental problems in the canal. In this study, the periphyton community and environmental factors on the left and right banks of the canal in the algal growing area were investigated and sampled six times (June, September, and November of 2019 and 2020). The succession pattern of the attached organism community in the artificial canal was analyzed, and the key factors affecting the algal community were analyzed using RDA and GAM. The results showed that the seasonal variability of the environmental factors was more significant than the spatial variability. A total of 114 taxa of periphytic algae were found, belonging to seven phyla and 69 genera, and mainly composed of Bacillariophyta. Species richness was ranked as Bacillariophyta (60 taxa), Chlorophyta (31 taxa) and Cyanobacteria (15 taxa), and higher in autumn than in summer. The dominant taxa were *Cymbella* sp., *Fragilaria* sp., *Navicula* sp. and *Diatoma* sp. The abundance of periphytic algal varied from 0.07 × 10^5^ to 8.99 × 10^5^ ind./cm^2^, with trends similar to that of species richness. The redundancy analysis and generalized additive model showed that water temperature and nutrient concentration were the key factors influencing the structure of the algal community, followed by discharge rate and velocity, which were the determinants of the spatial and temporal patterns of the algal community. In view of the influence of discharge and velocity on the structure of algal communities, it is suggested that ecological scheduling could be used to regulate the structure of the algal community on the canal wall in the operation of later water division projects to ensure the safety of water division.

## 1. Introduction

Periphytic algae are an important chemical regulator in aquatic ecosystems [1]. Studies have shown that during growth they can absorb not only N and P, but also heavy metals and other chemical elements in the aquatic ecosystem [2,3,4]. Periphytic algae are sensitive to environmental changes and their growth sites are relatively fixed. Hence, their biomass, biodiversity and community structure are important biological indicators for water quality monitoring [5,6]. However, excessive proliferation of periphytic algae may lead to a series of water environment problems [7]. Abnormal proliferation of algae might increase the risk of algae bloom. When the periphytic algae dies, they will be washed away and will be decomposed into organic debris particles. Under the action of flocculation, the organic debris particles and the suspended sediment particles bind to each other to form granular algae residues. The particles of these algae residues diffuse with the water, thus affecting the safety of water quality [8].

The Middle Route Project (MRP) of the South-to-North Water Diversion (SNWD) Project is the largest inter-basin water division project in the world. It is a major strategic basic project to realize the optimal allocation of water resources in China, promote sustainable social development, and ensure the improvement of people’s livelihoods. Since the water supply in 2014, it has become the lifeline, ecological line, and economic line for 140 million people in water-receiving areas. Its water quality status has always attracted special attention because it provides domestic and industrial water to northern China, and the water quality in the MRP is required to meet the Grade II Chinese Environmental Quality Standard for Surface Water (GB3838-2002). The MRP is primarily an open channel. The slope and bottom of the canal are made of concrete. There were few effective sun shelters such as trees on both sides of the canal. The canal water is clear and transparent, and sunlight passes through the canal bottom during the day, providing sufficient light for algae growth. The aquatic ecosystem of the canal is at the initial stage of formation. Although the nutrient content of the canal is relatively dynamic and stable, previous studies have found that a large number of periphytic algae bred on the canal wall of the local canal section at a specific time [8]. Algae residues formed by rapid growth of algae diffuse in the aquatic ecosystem and are easy to deposit at the sudden expansion sections such as sluice and diversion sluice, which affect the discharge and operation safety of water supply. In recent years, there have been some related studies on the relationship between the bacterioplankton [9], the phytoplankton [10,11] and the phytoplankton-water quality relationships [12] in the MRP. However, there are few studies on the community structure of periphytic algae in the canal of the MRP.

The main aim of the research was to determine the pattern of periphytic algae community in terms of time and space, as it may have an impact on water quality. In order to understand the succession pattern of the periphyton community and the response mechanism to environmental factors such as hydrological regulation and nutrients in the canal of the MRP in Henan province, the community structure of periphyton and environmental factors were investigated six times from 2019 to 2020, including June, September, and November, along the three canal sections of Diaohe, Fangcheng and Yuzhou according to the ecological location of the canal. The objectives of this study were to answer the following questions. (1) How does the periphyton community change in response to hydrological conditions and seasonal changes? (2) What are the key factors that influence the periphytic algae community in the MRP? The answers to these questions could help us to reveal the cause of periphyton accumulation, which may provide a scientific theoretical basis of ensuring the safe operation and sustainable management of the MRP.

## 2. Materials and Methods

### 2.1. Study Site and Sampling Period

The 1432 km MRP of SNWD (32.68°~39.99°N, 111.72°~116.27°E) went into service in 2014 and transferred 46 billion m^3^ water from the Danjiangkou Reservoir to Henan and Hebei Provinces, and Beijing and Tianjin Municipalities. The main channel is high in the south and low in the north, and the water conveyance mode is mainly an open channel and a partial pipe culvert. The designed discharge at the channel head is 350 m^3^/s, and the increased discharge is 420 m^3^/s. The discharge in the canal is continuous throughout the year to accommodate the water demands of a large population, and the water transportation volume tends to fluctuate with the amount of artificial withdrawal. 

**Figure 1:**
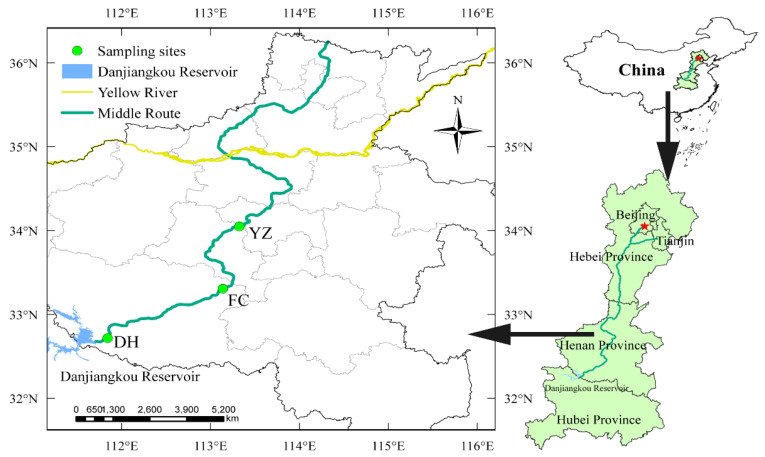
Distribution of sampling sites along the canal of the Henan section of the MRP of SNWD, China.

The Henan section spans the subtropical monsoon humid climate zone and temperate monsoon climate zone, characterized by four distinct seasons, simultaneous rain, and heat. The results of previous investigations showed that the areas with strong algae growth were all located in Henan Province. The areas where the main algae grow vigorously bend near the head of the main canal, the relatively flat and straight section in Fangcheng County of Nanyang City, the right bend in Yexian County of Pingdingshan City, and the relatively flat straight section in Yuzhou city in Pingdingshan City. Based on the preliminary survey of algae growth and the ecological location characteristics of the canal, three sampling points were selected from the south to the north along the Henan section canal [13]. The sampling points of Diaohe (DH) canal section in Dengzhou City, Fangcheng (FC) canal section in Nanyang City, and canal section in Yuzhou (YZ) City were selected as sampling sites, respectively (Figure 1).

Samples of both water and periphytic algae were collected at the beginning of each month in summer (June), early autumn (September) and late autumn (November), from June 2019 to November 2020.

### 2.2. Algae Sample Collection and Identification

The collection of periphytic algae is according to the sampling method of EPA [14], and was conducted in the areas where the algae grew on the slope of the canal at each site (10–80 cm from the water surface). An area of 400 cm^2^ was randomly selected, and a knife and brush was used to sample the algae. The samples were washed into a wide-mouth plastic bottle with 500 mL distilled water, and fixed with 4% formalin for identification. The samples were shaken well, and 0.1 mL of the sample was placed in the counting frame. Species identification [15,16,17] and counting were carried out under a microscope at 400× magnification. The number of cells per unit area was converted from the concentration times, and the number of cells was calculated as the abundance of the algae (ind./m^2^) [18]. The biomass of the algae was calculated according to the “Freshwater Plankton Research Methods” [19], and the unit was mg/m^2^.

### 2.3. Physical and Chemical Parameters Collection and Determination

According to the standard methods [20], water chemistry samples were collected at 0.5 m below the water surface at each sample site with a column water collector, and each sample was collected three times in parallel, sealed, and transported back to the laboratory for measurement. A portable multi-parameter water analyzer (YSI Professional Plus, Yellow Springs, USA) was used to determine the water temperature (WT), pH, turbidity (Tur), electrical conductivity (Cond), oxidation-reduction potential (ORP), and dissolved oxygen (DO). Discharge, velocity of flow (Vel) and depth were provided by the management office of the MRP.

Total phosphorus (TP) was analyzed by ammonium molybdate spectrophotometric method. Total nitrogen (TN) was measured by the alkaline potassium persulfate digestion-UV spectrophotometric method. Ammonia nitrogen (NH_4_^+^-N) was determined by Nessler’s Reagent Spectrophotometry. Nitrate nitrogen (NO_3_^−^-N) was determined by UV spectrophotometry. Chemical oxygen demand (COD_Mn_) was determined by a permanganate index. The determination methods were based on the Standard Methods for the Examination of Water and Wastewater in China [21].

### 2.4. Data Collation and Analysis

#### 2.4.1. Dominance Index

The McNaughton dominance index was used to determine the composition of dominant species, y > 0.02 then the dominant species (y) equation was as follows (Equation (1)).

y = (n_i_/N)f_i_ (1)

where f_i_ is the frequency of occurrence of the specie i at each point, n_i_ is the total number of individuals of species i, N is the total number of species. 

#### 2.4.2. Statistical Analysis

The relevant data were collated using Excel 2019 (Microsoft, Redmond, WA, USA). The spatial distribution of sample sites was plotted using ArcGIS 10.7 (ESRI Redlands, Redlands, CA, USA). Hydrological variability was plotted using Origin 9.0 software (Origin Lab, Northampton, MA, USA). One-way analysis of variance (ANOVA) of environmental factors among the different sampling sites with a significance level of *p* < 0.05 was used in SPSS 25 (IBM, Chicago, IL, USA). 

The principal component analysis (PCA) was applied to analyze the spatial and temporal variation in environmental factors among sites. The main environmental factors were standardized prior to all analyses. Correlation heatmap analysis was used to describe the relationship between environmental factors and dominant species. To choose a suitable model to fit the periphyton-environment relationship in the MRP canal, detrended correspondence analysis (DCA) of dominant species abundance data was used to determine whether linear or unimodal ordination methods should be applied. The dominant species of the algae were divided into two groups, one including diatoms and other including Chlorophyta, Cyanobacteria and Euglenophyta. The DCA results showed that the longest lengths were less than 3, and the linear redundancy analysis (RDA) could better explain the species-environment relationship. The above analyses were carried out in R (version 3.6.2) software (The R Programming Language, The University of Auckland, New Zealand) using vegan package [22], pheatmap package [23], and ggplot2 package [24].

Generalized additive models (GAMs) have been used to reveal the nonlinear relationship between response variables and explanatory variables in a highly flexible non-parametric smoothing method [25]. The GAM was used to analyze the effects of environmental variables on the biomass of algae in the Henan section of the MRP. The GAM equation was constructed using 10 environmental factors (WT, Tur, Cond, ORP, DO, TP, TN, NH_4_^+^-N, NO_3_^−^-N, and COD_Mn_) as the explanatory variables, and biomass as the response variable (Equation (2)).

Biomass = s(factor) + ε(2)

where Biomass indicates periphytic algae biomass, s denotes regression spline fit smoothing function, ε is a random error term. All GAMs were executed using the mgcv package in R program [26].

## 3. Results

### 3.1. Analysis of Hydrological and Environmental Factors in the MRP

#### 3.1.1. Dynamic Changes of Hydrological Factors

**Figure 2:**
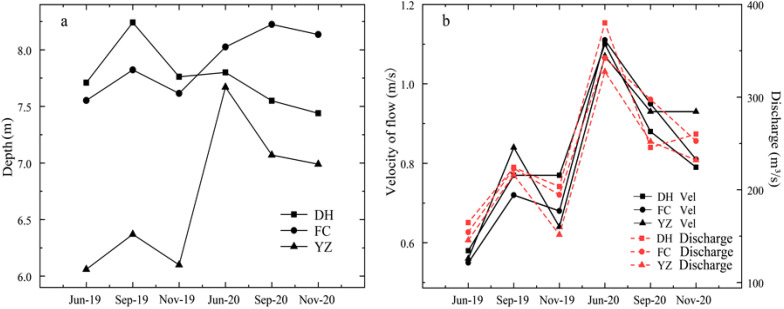
Hydrological changes in the Henan section of the MRP of SNWD: (**a**) Depth; (**b**) Flow velocity and discharge.

The hydrological changes in DH, FC, and YZ from June 2019 to November 2020 are shown in Figure 2. The average depth in DH, FC and YZ were 7.75 m, 7.89 m, and 6.71 m, respectively (Figure 2a). The water depth fluctuated significantly during the survey period, with the minimum water depth in June 2019 and the maximum water depth in June 2020, which was consistent among the sampling sites (Figure 2a). The discharge of the MRP was at a minimum of 145.66 m^3^/s in June 2019 and reached a maximum of 390.48 m^3^/s in June 2020. The MRP was at a high discharge (350 m^3^/s) for the first time in August 2019, and at a higher discharge (420 m^3^/s) for the second time from May to June 2020. The variation in Vel was from 0.55~1.11 m/s, with an annual average value of 0.83 m/s, a trend which was the same as that of discharge. (Figure 2b).

#### 3.1.2. Principal Component Analysis of Environmental Factors

**Figure 3:**
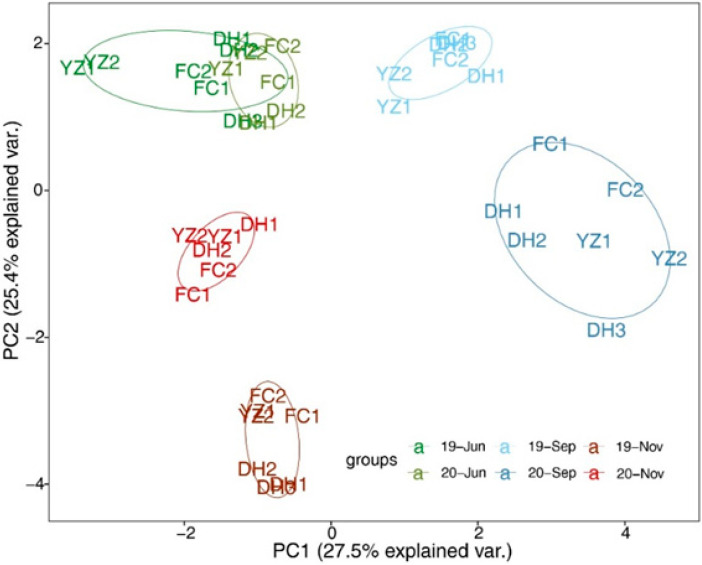
PCA of water environmental factors in the Henan section of the MRP of SNWD. (1 represents the left bank of the canal and 2 represents the right bank of the canal, 3 represents the bend section of the canal).

The results of PCA analysis showed that the PC1 axis explained 27.5% and the PC2 axis explained 25.4% of the variation (Figure 3). The analysis revealed the temporal variability of the environmental factors was greater than the spatial variability, indicating that the seasonal variability of the environmental factors in the samples was more evident. The environmental factors in June 2019 and June 2020 were relatively similar, the environmental factors in September 2019 and September 2020 were different, whereas the environmental factors in November 2019 and November 2020 were different.

#### 3.1.3. Physical and Chemical Properties of the Canal

From June 2019 to November 2020, the measured physicochemical factors of the MRP showed that the concentrations of physicochemical parameters displayed certain monthly variations (Table 1). The WT ranged from 14.5 to 28.1 °C, and peaked in September. The DO in June and November was significantly higher than that in September (*p <* 0.05). The COD_Mn_ concentration ranged from 1.3 to 3.1 mg/L, and its maximum value occurred in September. The TN concentrations ranged from 0.94 to 1.69 mg/L, with a mean value of 1.13 mg/L, the lowest in June and the highest in September (*p <* 0.05). NO_3_^−^-N concentrations in November were significantly higher than those in the other months (*p <* 0.05). TP concentrations ranged from 0.01 to 0.03 mg/L, with June significantly higher than in September and November. ORP was significantly different among months (*p <* 0.05). Other parameters were not significantly different in the three months. During the research period, there were no obvious differences in the spatial distribution of the physical and chemical indices.

### 3.2. Community Characteristics of Algal Species along the Canal

#### 3.2.1. Species Composition of Periphytic Algae along the Canal

**Figure 4:**
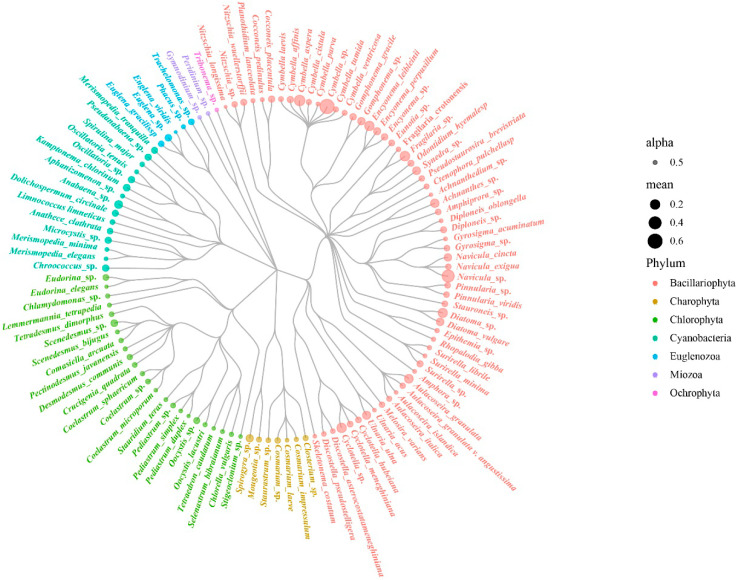
Evolutionary tree of species of algae in the Henan section of the MRP of SNWD.

From June 2019 to November 2020, 114 taxa of periphytic algae were identified in the Henan section of the MRP. These belonged to seven phyla, 69 genera and 72 species (including varieties) (Figure 4). Bacillariophyta (diatoms) were the most abundant phylum in terms of cell density, with 60 species (varieties) accounting for 52.63% of the total number of species. The next most common species was Chlorophyta (green algae), with 31 taxa (21.06%). Cyanobacteria, with 15 species (13.16%). Most Cyanobacteria detected were filamentous. Euglenozoa, Miozoa and Ochrophyta accounted for 6.14%, 4.39% and 0.87%, respectively.

**Figure 5:**
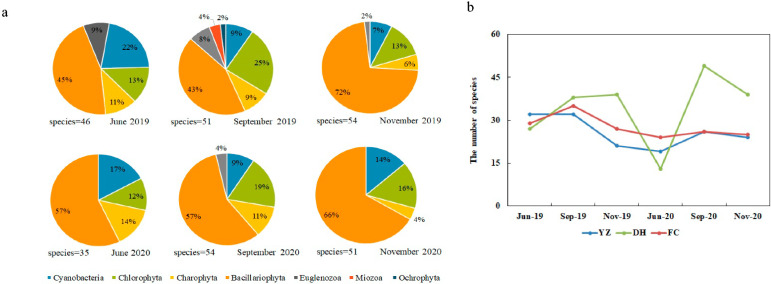
Spatial and temporal variation in species richness of periphytic algae by phyla in the sampling sites of MRP of SNWD: (**a**) Temporal variation in percentage composition of species richness during the sampling periods; (**b**) Temporal variation in total species richness during the sampling periods.

On a spatial scale, the number of periphytic species was higher at DH (81 species), followed by FC (64 species), and YZ (61 species). All the sampling sites were dominated by diatoms, including *Cymbella* sp., *Synedra* sp., *Navicula* sp. and *Diatoma* sp. The Charophyta were mainly *Cosmarium* sp. and *Spirogyra* sp. On a temporal scale, the fewest species were present in June 2020 (35 species), and the species number was comparable at other times (Figure 5b). The species in the MRP presented in the following descending order: Bacillariophyta, Chlorophyta, Cyanobacteria. During the study period, 58 species of six phyla were found in June, with half of the diatom species and a quarter of the Chlorophyta and Cyanobacteria each. In September, the proportion of diatoms remained the same, while the number of Chlorophyta increased to one-third and the number of Cyanobacteria decreased. In November, the number of diatom species increased to two-thirds, the proportion of Chlorophyta was the same as in June, and the proportion of Cyanobacteria decreased. The results showed that the community structure of the MRP canal has spatial and temporal heterogeneity. 

#### 3.2.2. Changes in Abundance of Algae in Canals

**Figure 6:**
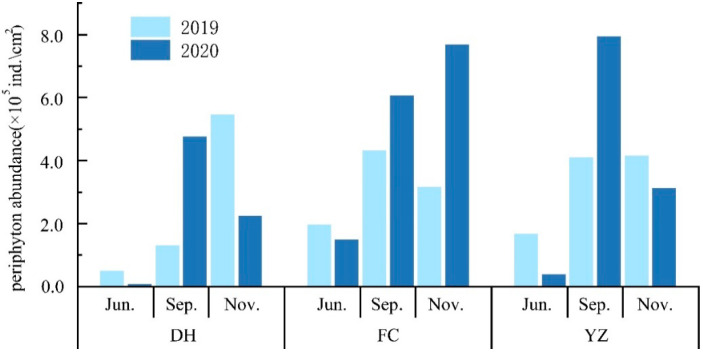
Temporal variation in abundance of algae in the Henan section of the MRP of SNWD.

From June 2019 to November 2020, the abundance of periphytic algae in the Henan section of the MRP varied from 0.07 × 10^5^ to 8.99 × 10^5^ ind./cm^2^ with a mean value of 3.24 × 10^5^ ind./cm^2^. The highest periphyton abundance (7.93 × 10^5^ ind./cm^2^) was observed in YZ, while the lowest abundance (0.84 × 10^5^ ind./cm^2^) was observed in DH. The average abundance of algae in FC was the highest at 4.12 × 10^5^ ind./cm^2^, followed by YZ (3.56 × 10^5^ ind./cm^2^), and DH (2.35 × 10^5^ ind./cm^2^) (Figure 6). There were interannual differences and temporal variations in algae abundance. In June 2019, the abundance of all stations was higher than that of the same period in 2020, whereas in September 2019, the abundance of all stations was lower than that of the same period in 2020. Maximum average abundance of periphyton 4.5 × 10^5^ind./cm^2^ was observed in September. November ranked second after September, with an average abundance of 4.5 × 10^5^ind./cm^2^. The mean abundance of algae was the lowest in June (0.97 × 10^5^ ind./cm^2^). Analysis of variance (ANOVA) showed that the abundance of algae was significantly different at different time scales (*p* < 0.05), but not in spatial distribution (*p* < 0.05).

#### 3.2.3. Dominant Species

The periphyton community of the Henan section of the MRP was identified 27 dominant species (Table 2), with *Cymbella aspera* (dominance 0.709) being the first dominant species. The periphyton communities of all sample sites were dominated mainly composed of diatoms, including *Cymbella* sp., *Synedra* sp., *Navicula* sp., and *Diatoma* sp. The dominant Chlorophyta were mainly *Cosmarium* sp. and *Spirogyra* sp. In June, diatoms, Cyanobacteria, Chlorophyta and Euglenophyta were the dominant groups. Among them *Anabaena* sp. and *Dolichospermum circinal* of the Cyanobacteria and *Diatoma* sp. of the Diatoms were the unique dominant species in June. The dominant groups in FC were similar in YZ, and *Euglena* sp., *Oscillatoria* sp., *Aulacoseira granulata* were dominant only in DH. Diatoms, Cyanobacteria and Chlorophyta were the dominant groups in September and November. Among them, *Chroococcus* sp., *Microcystis* sp., *Encyonema* sp., *Diploneis* sp. and *Achnanthes* sp. were the dominant species in September. *Encyonema* sp. and *Achnanthes* sp. were dominant in DH, FC and YZ, *Chroococcus* sp. was dominant in FC and YZ, *Microcystis* sp. was dominant only in FC, and *Diploneis* sp. was dominant only in DH. *Pseudanabaena* sp. was the dominant species in YZ only in November.

### 3.3. Relationship between Community Structure and Environmental Factors of Algal Species

#### 3.3.1. Relationship between Community Structure and Environmental Factors

**Figure 7:**
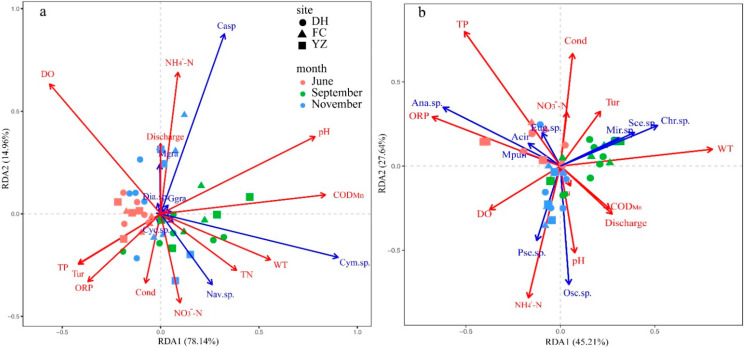
RDA ordination of algae community and environmental factors in Henan section of MRP of SNWD: (**a**) Cyanobacteria, Chlorophyta and Euglenophyta; (**b**) Bacillariophyta.

RDA was performed to reveal the relationship between environmental changes and periphyton community structure in a water body. The RDA ordination diagram including 11 environmental factors and the dominant species of diatoms is presented in Figure 7a. RDA axes 1 and axes 2 explained 45.21% and 27.64% of the total variation in the periphyton -environment relationships, respectively. Monte Carlo permutation tests showed that WT (*F* = 5.00, *p <* 0.001), TP (*F* = 5.09, *p <* 0.01), ORP (*F* = 3.93, *p <* 0.01) and NH_4_^+^-N (*F* = 2.94, *p <* 0.05) were the main environmental factors jointly driving the succession of the first group of algal communities. Cyanobacteria (*Chroococcus* sp., *Microcystis* sp.) and Chlorophyta (*Scenedesmus* sp.) were positively correlated with the WT and Tur. *Anabaena* sp., *Merismopedia punctata* and *Euglena* sp. were positively associated with TP and ORP, and negatively associated with COD_Mn_ and discharge. *Oscillatoria* sp. and *Pseudanabaena* sp. were positively correlated with NH_4_^+^-N concentration and pH (Figure 7a).

The RDA ordination diagram including 11 environmental factors and dominant species of nondiatoms, is presented in Figure 7b. The first two explained 93.1% of the total variability in the periphyton-environment relationships (RDA1 = 78.14% and RDA2 =14.96%). Monte Carlo permutation tests showed that COD_Mn_ (*F* = 20.18, *p <* 0.001), pH (*F* = 17.61, *p <* 0.001), DO (*F* = 9.19, *p <* 0.001), WT (*F* = 7.56, *p <* 0.01) and TP (*F* = 4.03, *p <* 0.01) were the most significant environmental factors influencing the changes in the dominant diatom community. *Cymbella aspera* was positive correlated with NH_4_^+^-N. *Cymbella* sp. showed a positively association with COD_Mn_, WT, and TN. *Aulacoseira granulate* and *Diatoma* sp. were positively correlated with discharge and NH_4_^+^-N. *Navicula* sp. was positively correlated with WT and NO_3_^−^-N, and negatively correlated with DO (Figure 7b).

**Figure 8:**
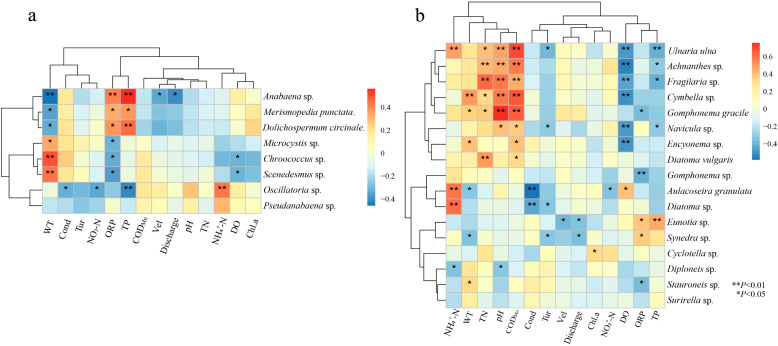
Heatmap of correlation between dominant species of periphytic algae and environmental variables: (**a**) Cyanobacteria, Chlorophyta and Euglenophyta; (**b**) Bacillariophyta.

To further understand the influence of environmental factors on the composition of the dominant algae community, correlation analysis was performed between the abundance of dominant species and environmental factors. As shown in Figure 8a, *Chroococcus* sp. and *Scenedesmus* sp. were positively correlated with WT (*p <* 0.05), while *Anabaena* sp. was negatively correlated with WT (*p <* 0.05). *Anabaena* sp., *Dolichospermum circinalis* and *Merismopedia tranquilla* showed a positive association with TP (*p < 0*.05), *Oscillatoria* sp. showed negative correlations with TP (*p <* 0.01), conductivity and with NO_3_^−^-N (*p <* 0.01), as well as positive correlation with NH_4_^+^-N (*p <* 0.05). *Anabaena* sp. showed a significant negative correlation with discharge and Vel (*p <* 0.05).

Most of the diatom taxa were significantly correlated with many environmental factors Figure 8b, *Synedra* sp., *Aulacoseira granulate* and *Diatoma* sp. showed significant positive correlations with NH_4_^+^-N (*p <* 0.05). *Eunotia* sp. was negatively correlated with discharge and Vel (*p <* 0.05). There was a negative correlation between *Synedra* sp. Discharge (*p <* 0.05). The abundance of other dominant diatom taxa was not significantly correlated with discharge and Vel. *Cymbella* sp. was positively correlated with WT (*p <* 0.01). *Aulacoseira granulate* and *Synedra* sp. were negatively correlated with WT (*p <* 0.05). *Achnanthes* sp., *Fragilaria* sp. *Diatoma vulgareis* were positively correlated with the TN (*p <* 0.01). *Ulnaria ulna*., *Achnanthes* sp., *Fragilaria* sp., *Cymbella* sp., *Gomphonema gracile*, *Navicula* sp., *Diatoma* sp. and *Encyonema* sp. showed significantly positive associations with COD_Mn_. *Synedra* sp., *Achnanthes* sp., *Fragilaria* sp., *Cymbella* sp., *Navicula* sp. and *Encyonema* sp. were negatively correlated with DO levels. The results of the correlation analysis were consistent with the RDA results (Figure 7), proving the high reliability of the correlation analysis.

#### 3.3.2. Effect of Different Water Environment Factors on the Biomass of the Periphytic Algae

The results of the multi-factor GAM analysis of the MRP with environmental factors are shown in Table 3, with a coefficient of determination of 0.83 and an explanation of variance of 90.9%. The degrees of freedom of Cond, TP, ORP and COD_Mn_ were greater than 1, indicating that they were non-linearly related to the biomass of algae, whereas the degrees of freedom of WT, DO, Tur, TN, pH, NH_4_^+^-N and NO_3_^−^-N were all 1, indicating that they were linearly related to the biomass of algae. By comparing the F-values of the 10 environmental factors, they were ranked as follows: Cond > TP > NH_4_^+^-N > ORP > DO > COD_Mn_. The F-values of Cond, TP, NH_4_^+^-N and ORP contributed more to the model, with F-values of 18.22, 16.14, 14.91 and 14.15, respectively, which were significantly correlated with the biomass of algae (*p* *<* 0.001). DO and COD_Mn_ had different effects on biomass, with F-values of 11.06 and 4.29. DO was significantly correlated with biomass (*p <* 0.001) and COD_Mn_ was significantly correlated with biomass (*p* *<* 0.01).

**Figure 9:**
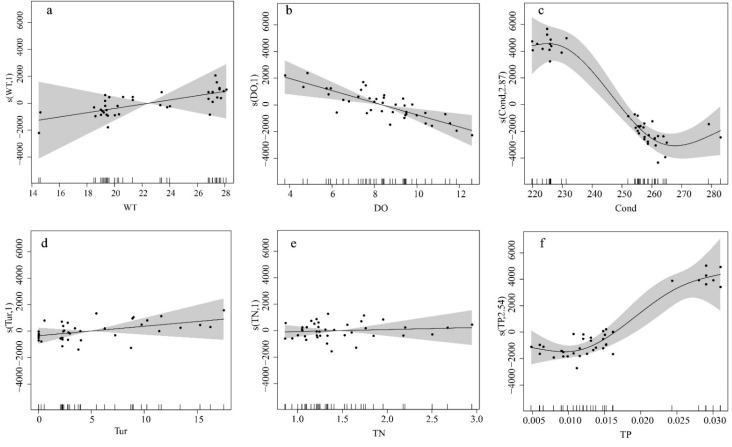

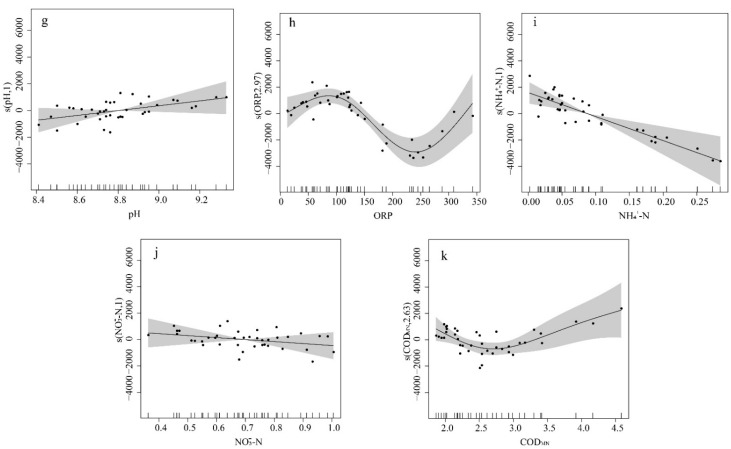
Illustration of the GAM for the effect of environmental factors on the biomass of the periphytic algae. (**a**) WT; (**b**) DO; (**c**) Comd; (**d**) Tur; (**e**) TN; (**f**) TP; (**g**) pH; (**h**) ORP; (**i**) NH4^+^-N; (**j**) NO_3_^−^-N; (**k**) CODMn.

The relationships for all explanatory variables and the response variable (biomass) are shown in Figure 9. The relationship between Tur, TN, pH and NO_3_^−^-N and the biomass was linear, with no significant changes in the biomass values as the values of the explanatory variables changed. Relative biomass increased in WT, TP, and COD_Mn_. The change in TP was obvious, when the TP concentration was less than 0.01 mg/L, the biomass increased slowly. However, the biomass rapidly increased when the TP concentration exceeded 0.015 mg/L. With an increase in WT, biomass increased more slowly. With an increase in COD_Mn_ concentration, the biomass first decreased and then increased, and the biomass appeared to monotonically increase when the COD_Mn_ concentration was more than 2.5 mg/L. Cond and ORP showed a trend of increasing and then decreasing, with a small increase in Cond from 220 to 230 μS/cm, a steep decrease from 230 to 260 μS/cm, and a slow increase when Cond exceeded 260 μS/cm. With an increase in ORP, the biomass effect value first increased and then decreased. These effects of DO and NH_4_^+^-N on biomass decreased with increasing values.

## 4. Discussion

### 4.1. Structure and Process of Algal Community

It has been shown that in natural, unpolluted rivers, the species composition of periphytic algae is mainly diatoms, with small amounts of Chlorophyta and Cyanobacteria [27]. During the study period, diatoms dominated the algae composition at each site of the MRP, with more than 50% of the algae, followed by Chlorophyta and Cyanobacteria (Figure 4). This was the same as the predominance of diatom species in the diatom community of rivers such as the Xiangxi River [28], a tributary of the Three Gorges Reservoir, and the Niyang River in Tibet [29]. The algal composition of the sampling sites in the Henan section of the MRP canal was similar to that of a naturally clean river, indicating that the water quality of the canal was relatively good. The periphyton community of the canal was similar to that of the Danjiangkou Reservoir [30], indicating that the delivery of water from the Danjiangkou Reservoir was also an important factor affecting the structure of the algal community. The regular pattern of periphyton community establishment was as follows: (i) particles or dissolved organic substances are electrostatically adsorbed on the substrate, (ii) bacteria grow, providing conditions for other organisms to attach and grow, (iii) pinnate diatoms are adsorbed on the parent substrate, (iv) upright, short-stemmed, or long-stemmed algae are rosetted or mucilaginous on the substrate, and (v) upright filamentous algae are attached [31,32]. Both Cyanobacteria and eukaryotic algae were present in the Henan section of the MRP, and some filamentous algae were common (Table 2). After 4 years of water transfer, the biological community of the MRP was relatively mature.

The general rule of evolution of the algal community is that diatoms living at low temperatures are dominant in spring and autumn, whereas diatoms, Chlorophyta and Cyanobacteria are dominant in summer. The species and quantity of algae are relatively low in winter [10]. Likewise, the seasonal variation in the algal community in the MRP canal was as follows: June was the beginning of summer, with diatoms, Chlorophyta and Cyanobacteria accounting for a quarter of the total, respectively. In September, at the end of summer and the beginning of autumn, the proportion of Bacillariophyta remained the same, whereas the number of Chlorophyta species increased and the number of Cyanobacteria decreased. In November, the number of diatom species increased in late autumn and early winter, the number of Chlorophyta and Cyanobacteria decreased (Figure 5). Other studies reported seasonal variation in the phytoplankton community in the canal, in agreement with our results [10]. Spatially, there was no significant difference between the communities of the sampling sites, which is consistent with the results of the PCA ranking analysis of the main environmental factors (Figure 3). Similar physical and chemical parameters would lead to a similar algal community [33,34]. In this study, we found that the communities at the sampling sites were similar. Although there were distances between them, the main environmental factors were not significantly different. This was probably due to the continuous and rapid flow of the water in the canal, and the algae migration and nutrients with the water during the process of the water transfer.

### 4.2. Influence of Environmental Factors on the Community of the Periphytic Algae

Algal growth is affected by hydrodynamic processes, nutrients, light and feeding, which determine the growth state, community structure and spatial distribution of algae [35]. The results of the RDA (Figure 7) and correlation analysis (Figure 8) showed that the main environmental factors that influenced the change in algal communities were WT, TP, COD_Mn_, NH_4_^+^-N, ORP, pH and DO. The influence of changes the algal community was similar to that of other aquatic bodies, indicating that the aquatic ecosystem in the MRP canal had some properties of natural water bodies.

The composition of periphytic algae is closely related to the water temperature. Temperature change directly affects the growth of algae by controlling the enzymatic reaction of algal photosynthesis or respiration, and indirectly affects the growth of algae by controlling the dissociation, dissolution, and decomposition of various substances [36]. Here, the WT in the canal ranged from 14.5 to 28.1 °C, the average WT in September was 27.35 °C, while in November was 19.38 °C, and the WT at each point in September was significantly higher than in the other two periods (Table 1). Chlorophyta had the highest species richness in September, whereas diatoms had the highest species richness in November (Figure 5). In general, Cyanobacteria and Chlorophyta are suitable for growth at higher water temperature, while diatoms were suitable for living at low water temperatures [37]. Correlation analysis showed that *Chroococcus* sp. and *Microcystis* sp. of Cyanobacteria and *Scenedesmus* sp. of Chlorophyta had a significant positive correlation with WT (*p* < 0.05). *Fragilaria* sp., *Navicula* sp., *Aulacoseira granulate* and *Synedra* sp. of the Bacillariophyta were negatively correlated with WT (*p* < 0.05) (Figure 8). The results of the GAM fit showed a positive correlation between the total biomass and water temperature (Figure 9). Therefore, water temperature influenced and determined the evolution of algal biomass and dominant taxa in the canal.

Nutrients are the material basis for algal survival and important environmental factors that affect algal changes. Among these, N and P are important factors that affect the cell density and community structure of algae [38]. The GAM results showed that the NH_4_^+^-N and TP concentrations were significantly correlated with biomass. Meanwhile, as the optimum phosphorus concentration for diatom growth was low (0.002~0.01 mg/L), the relative abundance of Cyanobacteria and Chlorophyta decreased, and diatoms gradually became dominant as the phosphorus concentration in the water column decreased [39]. A study of the South Finland River showed that TP was a key factor affecting diatom growth [40]. The concentration of TN in the canal was 0.9~1.69 mg/L and the concentration of TP was 0.01~0.03 mg/L. The nitrogen concentration was high, the phosphorus concentration was low, and the N/P ratio was much higher than 16, which showed that phosphorus limitation and nutrient condition were more suitable for the growth of diatoms [41]. The RDA results showed that COD_Mn_, which is an indicator of organic matter content in water, was also a nutrient factor affecting the diatom community in the MRP canal. This organic matter was probably mineralized by microorganisms, providing nutrients to periphytic algae. The study by Deng Peiyan et al. is consistent with a similar study in the Dongjiang River basin [42].

Discharge and velocity affect the composition of algal communities and the evolution of dominant taxa [43]. In general, Cyanobacteria and Chlorophyta are more suitable for growth in static or weakly turbulent waters, whereas diatoms can gain a greater proliferation advantage in flowing or turbulent environments [44]. The MRP is not a natural river, and the hydrodynamic conditions are influenced by the overlap of runoff processes and the regulation of the entire artificial canal. The canal has a continuous discharge throughout the year with velocities ranging from 0.5 to 1.11 m/s, with an average velocity of 0.88 m/s. It has a higher discharge than other water bodies; therefore, diatoms were the dominant taxa. Water disturbance significantly altered the algal community, resulting in a rapid decrease in biomass according to the study on the Danube floodplain [45]. The lowest number of algal species was found in June 2019, which had the lowest velocity in the canal. The abundance of *Anabaena* sp. was negatively correlated with velocity and discharge (*p* < 0.05), suggesting that the increase in velocity had an inhibitory effect on the growth of Cyanobacteria. Furthermore, the other study showed that the stability of filamentous Cyanobacteria colonization was susceptible to water flow conditions [46,47]. The correlation between diatom abundance and flow velocity was not significant. Compared with other periphytic algae, diatoms showed a higher tolerance to changes in the hydrological regime, and adapted more easily to different environmental conditions [48], especially those algae with large specific surface areas and strong adaptability to low light, such as *Cymbella* sp. [49]. Diatoms have good environmental adaptability and can easily become the dominant taxa in high-velocity water bodies. Therefore, it has become the dominant species on the studied section of the canal throughout the year. Compared with June 2019, the species richness and abundance of algae were significantly reduced during the high discharge of water transfer in June 2020, suggesting that hydrological changes affected the properties of the resident algal community. Hydraulic structures along the MRP have many basic conditions for algae control, which can effectively prevent and control algal blooms by optimizing operation and management to regulate hydrodynamic conditions and destroy the conditions for sudden algal blooms. It has been confirmed that changes in hydrodynamic conditions, such as discharge and velocity of flow could affect the spatial and temporal distribution of nutrients in the water body and change the dynamic response between algal growth and nutrients [50].

## 5. Conclusions

The main canal of the MRP is an engineered system in which the aquatic ecosystem is still in its early stages of formation. Despite the presence of algae in the canals, there is a lack of monitoring and research. Therefore, it is very important and urgent to investigate areas where algae grow vigorously and analyze the key factors affecting the growth of MRP algae. In this study, through monitoring and analysis of algae and hydrology, the changing pattern of algal community and the main factors affecting the changes in algal community were analyzed.

From June 2019 to November 2020, 114 taxonomic units of 114 species of periphytic algae belonging to seven phyla were identified in the Henan section of the MRP. Diatoms were the main species, followed by Chlorophyta and Cyanobacteria. The temporal effects of the changes in the structure of the host algal community were more significant than their spatial effects.

The RDA analysis showed that COD_Mn_, pH, DO, WT and TP were the most significant environmental factors influencing changes in the diatom community. WT, TP, ORP and NH_4_^+^-N were the main environmental factors that jointly drove the succession of Cyanobacteria and Chlorophyta communities. The GAM fitted the response mechanism of environmental factors on the biomass of the attached algae and showed that Cond, TP, NH_4_^+^-N and ORP were the main factors influencing the biomass of the periphytic algae in the Henan section of the MRP of the SNWD.

The turbulent water flow in the canal is not conducive to the proliferation of algae forming water blooms such as cyanobacteria and green algae, but it has a positive effect on the diversity and abundance of periphytic diatoms. This is advantageous in terms of the intended use of the water transported through the canal, which is largely used for human consumption.

## Figures and Tables

**Table 1:** Physicochemical parameters analyzed in the Henan section of the MRP of SNWD in different sampling periods.

Parameters	June	September	November
WT (°C)	20.32 ± 3.11a	27.35 ± 0.4b	19.38 ± 0.57a
pH	8.68 ± 0.12a	8.88 ± 0.29b	8.84 ± 0.15ab
DO (mg/L)	9.47 ± 1.01b	6.43 ± 1.38a	9.33 ± 1.83b
Cond (μS/cm)	262.21 ± 9.3b	258.08 ± 2.56b	224.97 ± 3.33a
ORP (mV)	203.65 ± 75.64c	58.87 ± 33.92a	143.62 ± 72.63b
Tur (NTU)	7.64 ± 6.16	4.8 ± 3.62	4.94 ± 3.99
NO_3_^−^-N (mg/L)	0.05 ± 0.02a	0.07 ± 0.05a	0.14 ± 0.1b
NH_4_^+^-N (mg/L)	0.71 ± 0.13	0.69 ± 0.09	0.69 ± 0.24
TN (mg/L)	1.14 ± 0.2a	1.69 ± 0.63b	1.51 ± 0.37ab
TP (mg/L)	0.022 ± 0.008b	0.012 ± 0.003a	0.011 ± 0.004a
COD_Mn_ (mg/L)	2.18 ± 0.26a	3.16 ± 0.73b	2.44 ± 0.33a

Note: Values were shown as arithmetic means ± standard deviations. Different lowercase letters represented significant differences by ANOVA at *p* < 0.05. Means with the same letter were not significantly different.

**Table 2:** Dominant species and dominant degree of periphytic algae in Henan section of MRP of SNWD.

Phylum	Dominant Species	Abbreviation	June	September	November
Cyanobacteria	*Anabaena* sp.	Ana sp.	0.333	—	—
	*Dolichospermum circinal*	Dcir	0.088	—	—
	*Chroococcus* sp.	Chr sp.	—	0.026	—
	*Merismopedia punctata*	Mpun	0.049	—	—
	*Microcystis* sp.	Mir sp.	—	0.034	—
	*Oscillatoria* sp.	Osc sp.	0.052	—	0.034
	*Pseudanabaena* sp.	Pse sp.	—	—	0.025
Chlorophyta	*Scenedesmus* sp.	Sce sp.	0.237	0.073	0.075
Bacillariophyta	*Achnanthes* sp.	Ach sp.	—	0.032	—
	*Cyclotella* sp.	Cyc sp.	0.336	0.077	0.107
	*Cymbella* sp.	Cym sp.	0.373	0.175	0.307
	C. *aspera*	Casp	0.677	0.904	0.546
	*Diatoma* sp.	Dia sp.	0.5	—	0.18
	D. *vulgareis*	Dvul	0.167	0.026	—
	*Diploneis* sp.	Dip sp.	—	0.022	—
	*Encyonema* sp.	Enc sp.	—	0.374	—
	*Eunotia* sp.	Eun sp.	0.022	—	—
	*Fragilaria* sp.	Fra sp.	0.18	0.087	0.14
	*Gomphonema* sp.	Gom sp.	0.026	—	—
	G. *gracile*	Ggra	—	0.024	—
	*Aulacoseira granulata*	Agra	0.166	0.024	0.169
	*Navicula* sp.	Nav sp.	0.349	0.274	0.287
	*Stauroneis* sp.	Sta sp.	—	0.025	—
	*Surirella* sp.	Sur sp.	—	0.02	—
	*Synedra* sp.	Syn sp.	0.06	—	0.027
	*Ulnaria ulna*	Uuln	—	0.059	0.1
Euglenophyta	*Euglena* sp.	Eug sp.	0.073	—	—

Note: —indicates dominance <0.02. Numbers indicates the dominance index.

**Table 3:** Fitting results of GAM between algal biomass and environmental factors in the MRP of SNWD.

Parameters	df	Ref.df	F	P
WT	1.000	1.000	0.778	0.388
DO	1.000	1.000	11.059	0.003 **
Cond	2.867	2.982	18.216	0.000 ***
Tur	1.000	1.000	1.336	0.261
TN	1.000	1.000	0.124	0.728
TP	2.541	2.824	16.143	0.000 ***
pH	1.000	1.000	2.435	0.134
ORP	2.974	2.999	14.152	0.000 ***
NH_4_^+^-N	1.000	1.000	14.912	0.001 ***
NO_3_^−^-N	1.000	1.000	0.83	0.373
COD_Mn_	2.629	2.893	4.287	0.025 *

Note: * indicated variable significant at 0.05 level, ** indicated variable significant at 0.01 level, and *** indicated variable significant at 0.001 level.

## Data Availability

Not applicable.

## References

[1] Liu J.K. (1999). Advanced Aquatic Biology.

[2] Lan B. (2011). Ecological Study of the Periphyton of Lake Erhai. Master’s Thesis.

[3] Lishani W., Wu N.C., Nicola F., Tenna R. (2022). Epiphytic biofilms in freshwater and interactions with macrophytes: Current understanding and future directions. Aquat. Bot..

[4] Liang X. (2007). Effect of Periphyton on Water Environment and Its Application in Water Quality Treatment. Ph.D. Thesis.

[5] Cattaneo A. (1987). Periphyton in Lakes of Different Trophy. Can. J. Fish. Aquat. Sci..

[6] Yi R., Cai D.S., Zhang Y.X. (2015). Benthic diatom assemblages distribution in Longjiang River, in relation to environmental factors. Environ. Sci. Technol..

[7] Lee Y.O., Park J.H., Park J.K. (2005). Microbial Characterization of Excessive Growing Biofilm in Sewer Lines Using Molecular Technique. J. Microbiol. Biotechnol..

[8] Wu D. (2019). Study on the Movement Characteristics of Algae Debris Particles. Master’s Thesis.

[9] Chen Z.J., Chen H.Y., Li Y.Y. (2017). Community structure and influencing factors of bacterioplankton in the Main Cancel of the Midline Project of South-to-North Water Division in sections of Henan Province. China Environ. Sci..

[10] Zhang C.M., Zhu Y.X., Song G.F., Mi W.J., Bi Y.H. (2021). Spatiotemporal pattern of phytoplankton community structure and its determining factors in the channel of the middle route of South-to-North Water Diversion Project. J. Lake Sci..

[11] Yi X., Lin J.Q., Jin Q., Lei X., Yuan R. (2020). A study on the phytoplankton community structure in the Diaohe River section of the Middle Route of the South-to-North Water Diversion Project in winter. Water Sci. Technol. Water Supply.

[12] Nong X.Z., Shao D.G., Shang Y.M., Liang J.K. (2021). Analysis of spatio-temporal variation in phytoplankton and its relationship with water quality parameters in the South-to-North Water Diversion Project of China. Environ. Monit. Assess..

[13] Zhu J., Lei X.H., Quan J., Yue X. (2019). Algae Growth Distribution and Key Prevention and Control Positions for the Middle Route of the South-to-North Water Diversion Project. Water.

[14] Peck D.V., Herlihy A.T., Hill B.H., Hughes R.M., Kaufmann P.R. (2006). Environmental Monitoring and Assessment Program: Surface Waters. Western Pilot Study: Field Operations Manual for Wadeable Streams. https://www.researchgate.net/publication/235943200_Environmental_Monitoring_and_Assessment_Program-Surface_Waters_Western_Pilot_Study_Field_Operations_Manual_for_Wadeable_Streams.

[15] Hu H.J., Wei Y.X. (2006). The Freshwater Algae of China: Systematics, Taxonomy and Ecology.

[16] Resources T.M.O.W. (2012). Map of Common Algae in Chinese Inland Waters.

[17] Shi Z.X. (2013). Flora Algarum Sinicarum Aquae Dulcis.

[18] Sun J., Liu D., Qian S. (1999). Study on phytoplankton biomass I. Phytoplankon measurement biomass from cell volime or plasma volume. Acta Oceanol. Sin..

[19] Zhang Z.S., Huang X.F. (1991). Research Methods of Freshwater Plankton.

[20] China M.E.A.E. (2002). Environmental Quality Standards for Surface Water. https://english.mee.gov.cn/Resources/standards/water_environment/quality_standard/200710/t20071024_111792.shtml.

[21] Administration S.E.P. (2002). Methods for Monitoring and Analysis of Water and Wastewater.

[22] Oksanen J., Blanchet F.G., Kindt R., Legendre P., Minchin P.R., O’Hara R.B., Simpson G.L., Solymos P., Stevens M.H.H., Wagner H. (2020). Vegan: Community Ecology Package. https://CRAN.R-project.org/package=vegan.

[23] Kolde R. (2019). Pheatmap: Pretty Heatmaps. https://CRAN.R-project.org/package=pheatmap.

[24] Wickham H. (2016). Ggplot2: Elegant Graphics for Data Analysis.

[25] Hastie T.J., Tibshirani R.J. (1990). Generalized Additive Models.

[26] Wood S.N. (2011). Fast Stable Restricted Maximum Likelihood and Marginal Likelihood Estimation of Semiparametric Generalized Linear Models. J. R. Stat. Soc. Ser. B.

[27] Feng T.Y., Song C., Chen J.Z. (2011). Environmental Indication Function of Aquatic Algae. Chin. Agric. Sci. Bull..

[28] Ja X.H., Wu N.C., Tang T., Cai Q.H. (2008). Spatiotemporal variation of epilithic algae in Xiangxi River system. Chin. J. Appl. Ecol..

[29] Liu H.P., Ye S.W., Yang X.F. (2013). Spatio-temporal dynamics of aquatic organism community and their relationships to environment in Niyang River, Tibet:2. periphytic algae. J. Lake Sci..

[30] Zheng B.H., Zhu J.Y., Xu X., Xin Y. (2018). Community structure of periphyton algae and water quality in the Danjiangkou Reservoir. J. Henan Norm. Univ..

[31] Hao B.B., Wu H.P., Cao Y., Xing W., Jeppesen E. (2017). Comparison of periphyton communities on natural and artificial macrophytes with contrasting morphological structures. Freshw. Biol..

[32] Casartelli M.R., Ferragut C. (2018). The effects of habitat complexity on periphyton biomass accumulation and taxonomic structure during colonization. Hydrobiologia.

[33] Rodríguez-Alcalá O., Blanco S., García-Girón J., Jeppesen E. (2019). Large-scale geographical and environmental drivers of shallow lake diatom metacommunities across Europe. Sci. Total Environ..

[34] Roder H.L., Olsen N.M.C., Whiteley M., Burmolle M. (2020). Unravelling interspecies interactions across heterogeneities in complex biofilm communities. Environ. Microbiol..

[35] Dunck B., Schneck F., Rodrigues L. (2016). Patterns in species and functional dissimilarity: Insights from periphytic algae in subtropical floodplain lakes. Hydrobiologia.

[36] Engle V.D., Summers J.K., Macauley J.M. (1999). Dissolved Oxygen Conditions in Northern Gulf of Mexico Estuaries. Environ. Monit. Assess..

[37] Trombetta T., Vidussi F., Mas S., Parin D., Mostajir B. (2019). Water temperature drives phytoplankton blooms in coastal waters. PLoS ONE.

[38] Zhu Y.Q., Jun L., Li Q., Zhang X.L. (2019). Seasonal variation of phytoplankton community and its relationship with environment in subtropical reservoirs: A comparison between two methods of functional groups classification. J. Appl. Ecol..

[39] Yang W., Deng D.G., Meng X.L., Zhang S. (2019). Temporal and Spatial Variations of Phytoplankton Community Structure in Lake Erhai, a Chinese Plateau Lake, with Reference to Environmental Factors. Russ. J. Ecol..

[40] Soininen J., Könönen K. (2004). Comparative study of monitoring South-Finnish rivers and streams using macroinvertebrate and benthic diatom community structure. Aquat. Ecol..

[41] Güsewell S. (2010). N:P ratios in terrestrial plants: Variation and functional significance. New Phytol..

[42] Deng P.Y., Zhang W., Wang X.T. (2015). The effects of water quality on epilithic diatoms communities of Dongjiang river basin. Acta Ecol. Sin..

[43] Wang M., Wu H., Ma J. (2004). Causes, and characteristics of the euterophication in large reservoirs in the Yangtze basin. Resour. Environ. Yangtze Basin.

[44] Li P.F., Gao Y., Zhang H.P. (2015). imulation experiment on the effect of flow velocity on phytoplankton growth and composition. J. Lake Sci..

[45] Pfeiffer T.Z., Mihaljević M., Maronić D., Stević F. (2015). The disturbance driven changes of periphytic algal communities in a Danubian floodplain lake. Knowl. Manag. Aquat. Ec..

[46] Flynn K.F., Asce M., Chapra S.C., Asce F. (2020). Evaluating Hydraulic Habitat Suitability of Filamentous Algae Using an Unmanned Aerial Vehicle and Acoustic Doppler Current Profiler. J. Environ. Eng..

[47] Wellnitz T., Poff N.L. (2012). Current-mediated periphytic structure modifies grazer interactions and algal removal. Aquat. Ecol..

[48] Bichoff A., Osório N.C., Dunck B., Rodrigues L. (2016). Periphytic algae in a floodplain lake and river under low water conditions. Biota Neotrop..

[49] Li Y. (2018). Spatial and Temporal Succession Characteristics of Phytoplankton Functional Groups and their Relationship with Environmental Factors in Dianshan Lake. Master’s Thesis.

[50] Yang Z.H., Yang S.C., Li D., Bai F.P. (2016). Numerical simulation of eutrophication and its test of ecological operation schedule in Xiaojiang River, the tributary of Three Gorges Reservoir. J. Lake Sci..

